# Supplementary material for: Disruption of the PDZ domain–binding motif of the dopamine transporter uniquely alters nanoscale distribution, dopamine homeostasis, and reward motivation
Source: J Biol Chem. 2021 Oct 29;297(6):101361. doi: 10.1016/j.jbc.2021.101361 (PMC8648841; doi:10.1016/j.jbc.2021.101361)
Supplement: Supplemental Figures S1–S4 [file mmc1.pdf]

Disrupted PDZ-domain binding motif of the dopamine transporter uniquely alters nanoscale distribution, dopamine homeostasis and reward motivation

Gunnar Sørensen[1,2], Mattias Rickhag[1], Damiana Leo[3,$], Matthew D. Lycas, Pernille Herrstedt Ridderstrøm[2], Pia Weikop[2], Jamila H. Lilja[1], Pedro Rifes[1], Freja Herborg[1], David Woldbye[1], Gitta Wörtwein[2], Raul R. Gainetdinov[4], Anders Fink-Jensen[2] and Ulrik Gether*[1]

SUPPORTING INFORMATION:

Figure S1. The DAT immunosignal is specific.

Figure S2. Figure S2. Quantitative analysis of the distribution of DAT and VMAT2 dSTORM localizations in DAT-AAA varicosities.

Figure S3. Figure S3. Quantitative real-time PCR (qRT-PCR) shows unchanged transcription of the genes encoding D_1R (*Drd1*) and D_2R (*Drd2*) in DAT-AAA mice.

Figure S4.
Figure S4. Operant naïve acquisition of 1.0 mg/kg/infusion cocaine.

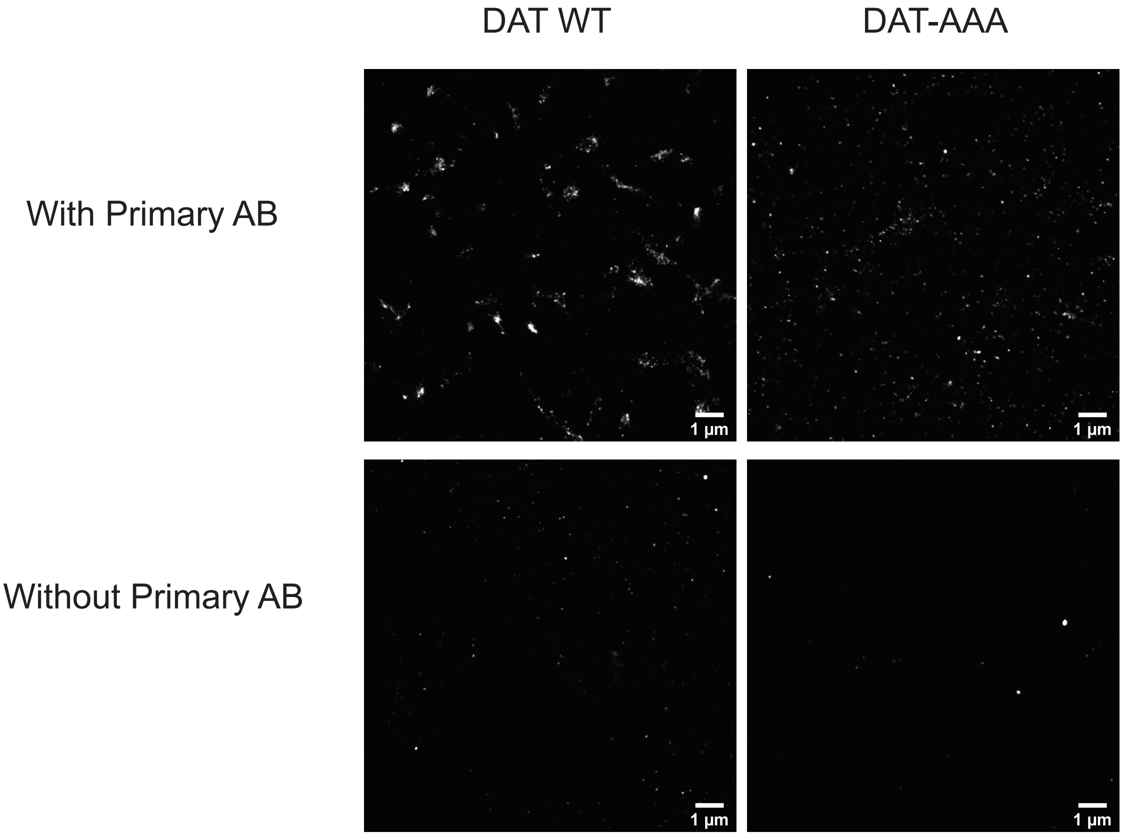

Figure S1. The DAT immunosignal is specific. *Upper panel*, Example dual color dSTORM image of striatal slice from WT (left) and DAT-AAA mouse (right) immunostained with primary MAB369 monoclonal antibody and subsequently labeled with Alexa 647-conjugated secondary antibody. *Lower panels,* Example dual color dSTORM image of striatal slice from WT (left) and DAT-AAA mouse (right) immunostained solely with secondary antibody.

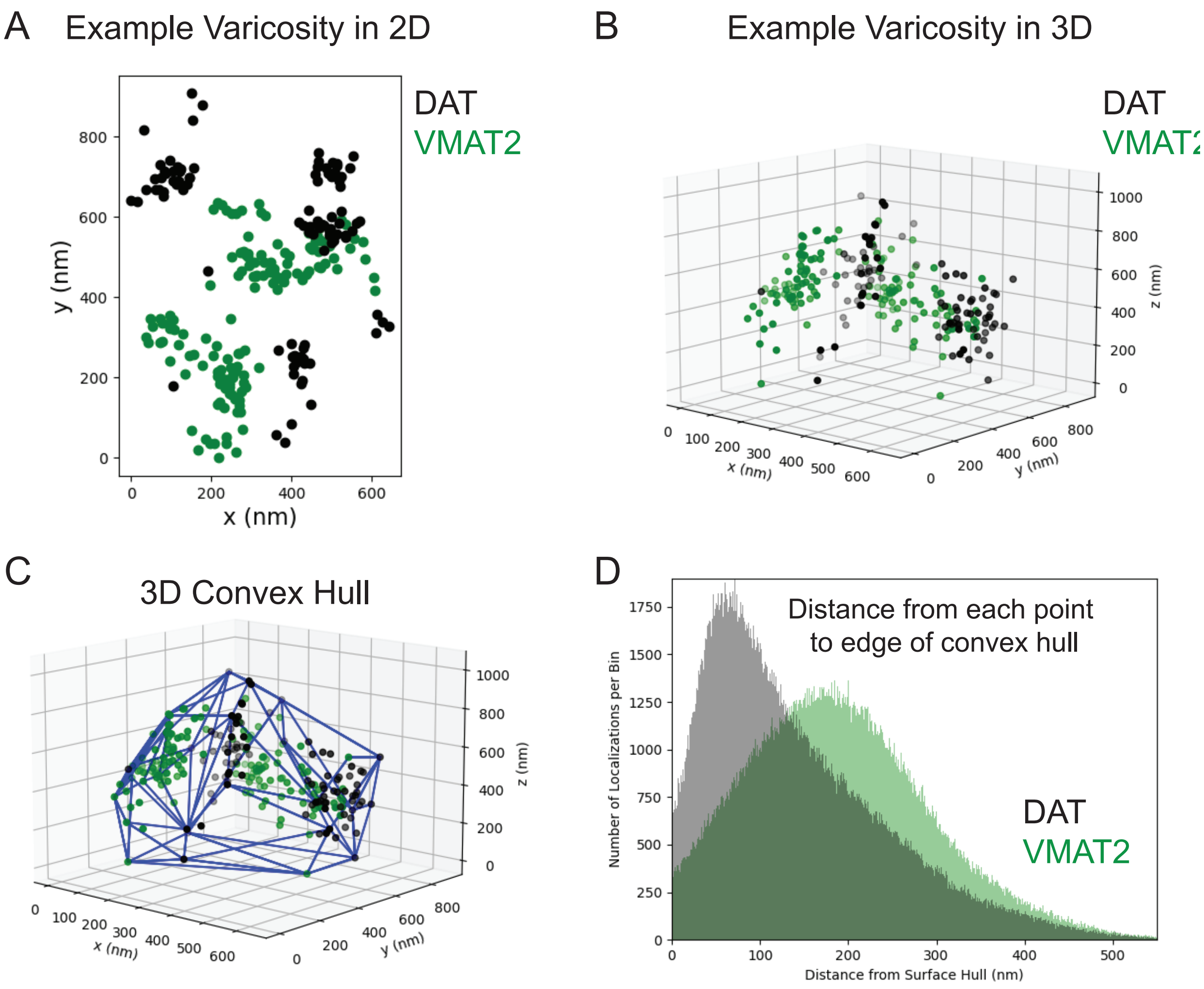

Figure S2. Quantitative analysis of the distribution of DAT and VMAT2 dSTORM localizations in DAT-AAA varicosities. *A,* Example varicosity from a dSTORM image for DAT and VMAT2 from a DAT-AAA mouse striatal slice. Shown in 2D. *B*, The same varicosity from A, displayed in 3D. Z positions identified by astigmatism (see Methods). *C*, A convex hull is applied to the total DAT and VMAT2 localizations present in the example varicosity. *D,* The distance from each localization to the convex hull of its varicosity was calculated and compiled into a histogram. The calculations shown are from the entirety of DAT-AAA varicosities isolated in the study (610.883 DAT localizations and 580.886 VMAT2 llocalizations taken from 1186 DAT-AAA varicosities). The majority of DAT-AAA localizations resides markedly closer to the likely localization of the plasma membrane (the convex hull) than the obligate intracellular VMAT2 localizations. Because the VMAT2 is localized in cytosol on synaptic vesicles that predictably would be close to the plasma membrane, the data support that the DAT-AAA localizations identified for the most part resides in the plasma membrane and not in an intracellular compartment. Unfortunately, we cannot reliably carry out the same analysis for WT. Since the analysis involves finding the z position based off the degree of ellipticity as compared to a measured reference standard, it is vital to have maximum spatial isolation between individual localization emissions in order to discern the lower intensity regions of a given PSF accurately. This was not possible for the WT DAT dataset because of the high density of dSTORM localizations.

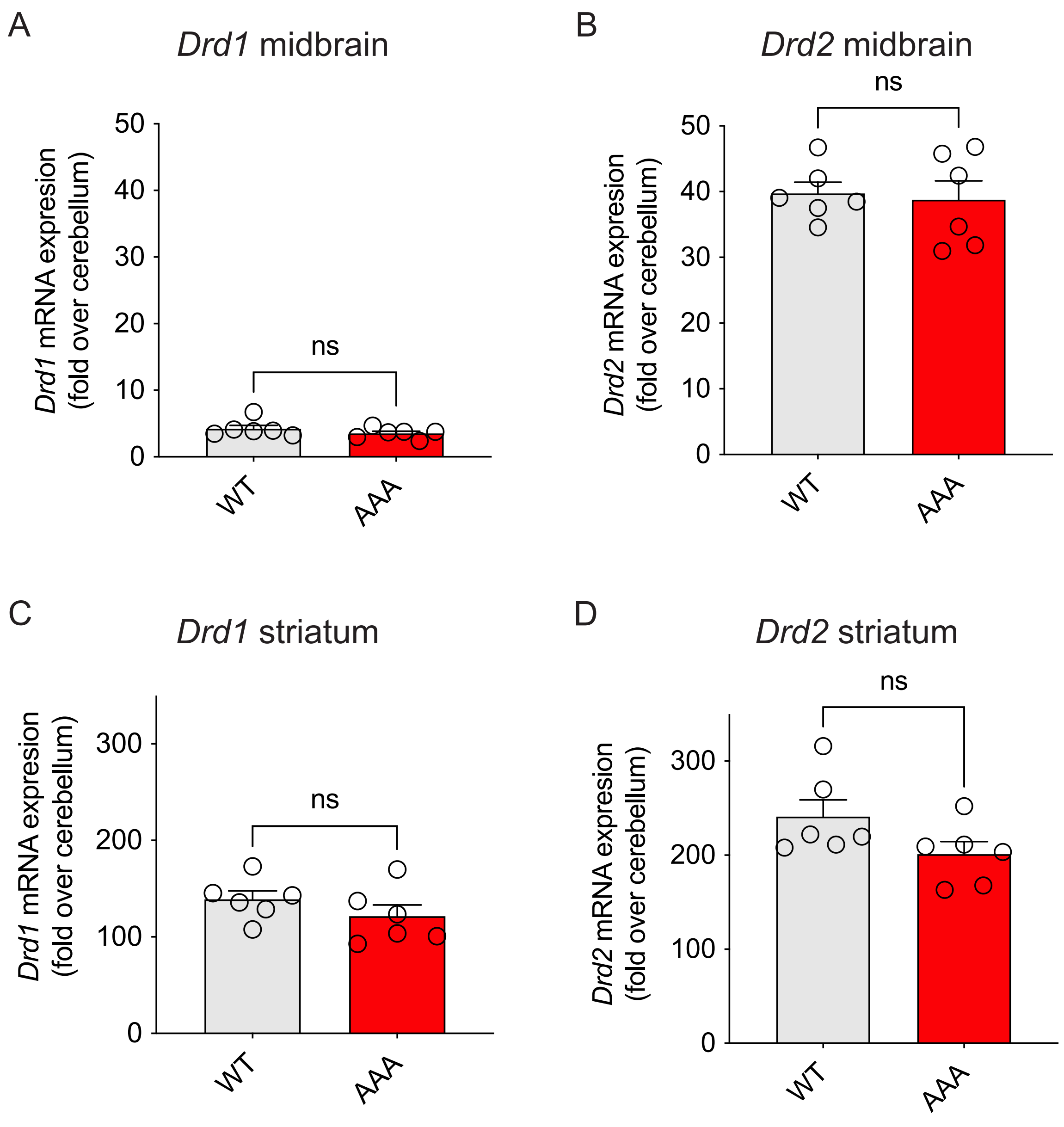

Figure S3. Quantitative real-time PCR (qRT-PCR) shows unchanged transcription of the genes encoding D_1R (*Drd1*) and D_2R (*Drd2*) in DAT-AAA mice. *A,* Comparison between WT and DAT-AAA of *Drd1* transcription in midbrain neurons (presynaptic expression). *B,* Comparison between WT and DAT-AAA of *Drd2* transcription in midbrain neurons (presynaptic expression). *C,* Comparison between WT and DAT-AAA of *Drd1*transcription in striatal neurons (postsynaptic expression). *D,* Comparison between WT and DAT-AAA of *Drd2* transcription in midbrain neurons (postsynaptic expression). Data are shown as fold change above reference tissue (cerebellum)= $2^{-(\Delta\Delta Ct)}$ (means±S.E., n=6). Data were analyzed by non-parametric Mann-Whitney test (ns, not significant).

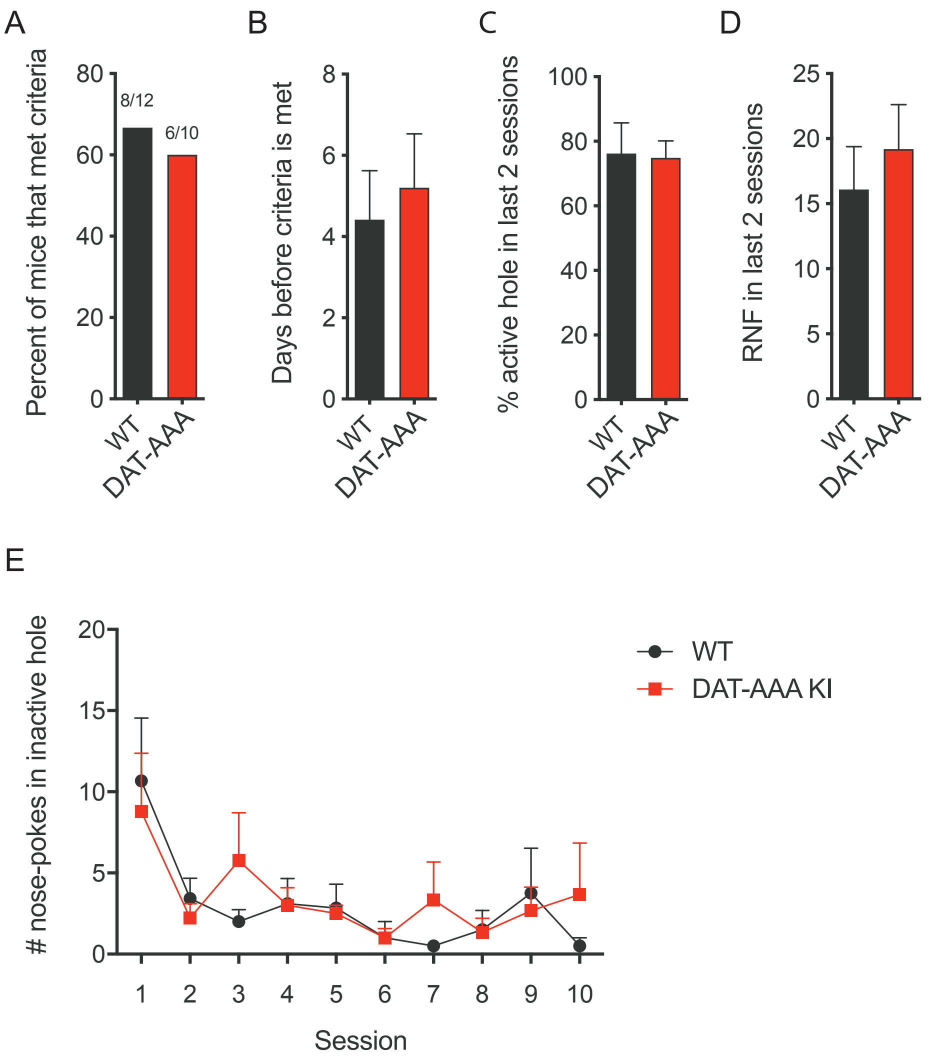

Figure S4. Operant naïve acquisition of 1.0 mg/kg/infusion cocaine. Operant naïve acquisition of 1.0 mg/kg/infusion showed no difference between DAT-AAA and WT mice in any of the criteria measured; *A,* Percent of mice that met criteria. *B,* Days before criteria was met. *C,* Percent nose pokes in the active nose poke hole at criteria. *D,* Number of reinforcers at criteria. *E,* Percent nose pokes in the active nose poke hole through-out all 10 sessions.
